# Inflammation in atherosclerosis: pathophysiology and mechanisms

**DOI:** 10.1038/s41419-024-07166-8

**Published:** 2024-11-11

**Authors:** Amir Ajoolabady, Domenico Pratico, Ling Lin, Christos S. Mantzoros, Suhad Bahijri, Jaakko Tuomilehto, Jun Ren

**Affiliations:** 1 Department of Biomedical Engineering, University of Alabama at Birmingham, Birmingham, AL 35294 USA; 2 Alzheimer’s Center at Temple, Lewis Katz School of Medicine, Temple University, Philadelphia, PA 19140 USA; 3 Shanghai Institute of Cardiovascular Diseases, Department of Cardiology, Zhongshan Hospital Fudan University, Shanghai, 200032 China; 4 National Clinical Research Center for Interventional Medicine, Shanghai, 200032 China; 5 Harvard Medical School, Harvard University, Boston, MA 02115 USA; 6 Diabetes Research Group, King Abdulaziz University, Jeddah, Saudi Arabia; 7 Department of Public Health, University of Helsinki, Helsinki, Finland; 8 Health Promotion Unit, Finnish Institute for Health and Welfare, Helsinki, Finland

**Keywords:** Chronic inflammation, Atherosclerosis

## Abstract

Atherosclerosis imposes a heavy burden on cardiovascular health due to its indispensable role in the pathogenesis of cardiovascular disease (CVD) such as coronary artery disease and heart failure. Ample clinical and experimental evidence has corroborated the vital role of inflammation in the pathophysiology of atherosclerosis. Hence, the demand for preclinical research into atherosclerotic inflammation is on the horizon. Indeed, the acquisition of an in-depth knowledge of the molecular and cellular mechanisms of inflammation in atherosclerosis should allow us to identify novel therapeutic targets with translational merits. In this review, we aimed to critically discuss and speculate on the recently identified molecular and cellular mechanisms of inflammation in atherosclerosis. Moreover, we delineated various signaling cascades and proinflammatory responses in macrophages and other leukocytes that promote plaque inflammation and atherosclerosis. In the end, we highlighted potential therapeutic targets, the pros and cons of current interventions, as well as anti-inflammatory and atheroprotective mechanisms.

## Facts

- The pathophysiology of atherosclerosis is closely linked with inflammation and its transition to chronic inflammation.
- Monocytes/macrophages are at the center of driving plaque inflammation in atherosclerosis with broad and complicated molecular mechanisms, which are not fully deciphered.
- Current molecular findings on atherosclerotic inflammation are less likely to be translated into the clinic.

## Open questions

- Why is the therapeutic translation of identified molecular targets in atherosclerotic inflammation limited?
- How to exploit some of the endogenous mechanisms activated within macrophages that appear to be anti-inflammatory and atheroprotective?
- Why the discovery of molecular mechanisms of inflammation in atherosclerosis should be prioritized over impetuous therapeutic interventions (often mistaken for innovative strategies)?

## Introduction: An overview of atherosclerosis

Atherosclerosis critically contributes to the onset and development of various forms of cardiovascular disease (CVD) including coronary artery disease and peripheral arterial disease [1–3]. Atherosclerosis is characterized by the thickening of arteries caused by the formation of plaques consisting of fatty acids, cholesterol, calcium, fibrin, cellular debris, and waste products in the subendothelium. This leads to the varying degrees of arterial stenosis, which may completely occlude the blood flow, causing hypoxia in vital organs such as the heart, brain, kidneys, pelvis, arms, and lower extremities [4–7]. As plaques grow, they become unstable and may rupture, leading to blood coagulation at the rupture site and further occluding downstream veins or arteries, a condition commonly known as thrombosis [7–10]. As atherosclerosis progresses, **myeloid cells** “(see the Glossary)” further increase the risk of plaque rupture, prompting myocardial infarction (MI) and stroke. These pathological events represent the leading complications of atherosclerosis with alarming mortality rates worldwide [11–13]. Notably, the majority of coronary plaques that rupture and cause MI are not the most stenotic but rather possess features that make them more vulnerable such as thin **fibrous caps** and a high content of activated macrophages [14].

In a recent examination of human carotid plaques from three different cohorts, histological and electron microscopic evaluation revealed that plaque ruptures mainly occurred in the most stenotic and proximal regions of carotid plaques [15]. These regions also displayed higher plaque vulnerability, thrombosis, and the proportion of inflammatory cells compared with distal regions [15]. Then, cutting-edge techniques including bulk and spatial RNA sequencing were performed on each region, and the results revealed differentially expressed genes in proximal and stenotic regions compared with distal regions [15]. These differentially expressed genes were associated with plaque ruptures and the degradation/remodeling of the extracellular matrix (ECM) in proximal and stenotic regions [15]. Among these genes was *MMP9*/MMP-9, the expression level of which was predominantly high in stenotic regions. Besides, Mendelian randomization analyses showed that *rs3918249* and *rs11699481* polymorphisms of *MMP9* gene were associated with the higher circulatory levels of MMP-9 and the risk of coronary atherosclerosis [15]. Hence, the site-specific transcriptomic analyses of carotid plaques can reveal genes/transcriptomes closely linked to the risk of plaque rupture.

**Fig. 1:**
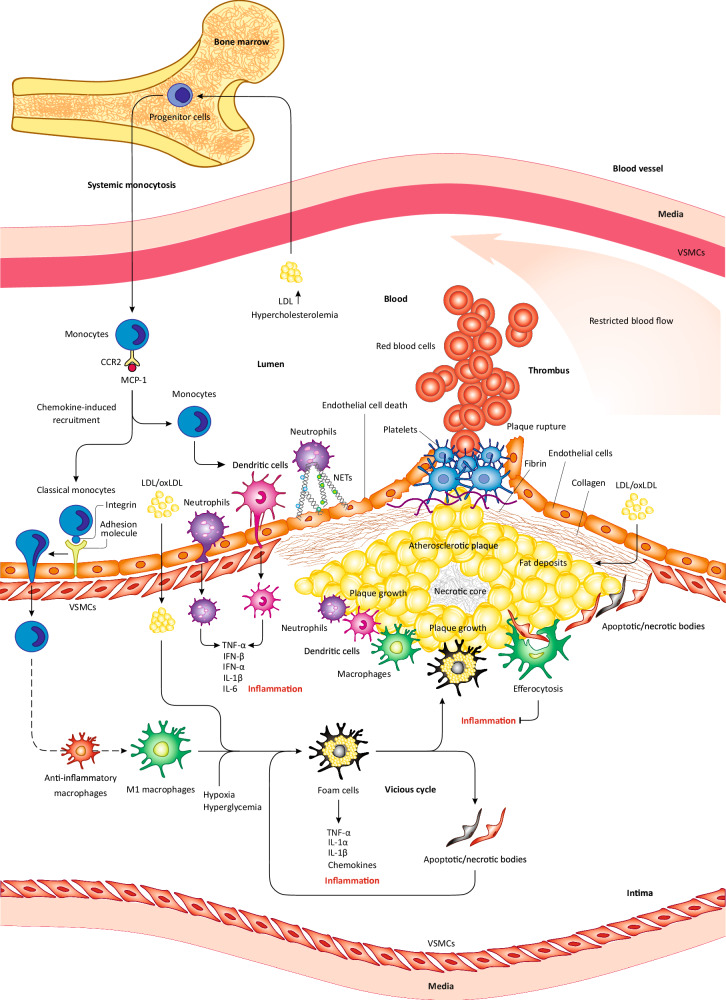
Atherosclerotic plaque, the infiltration of inflammatory cells, and inflammation. The excessive retention or oxidation of LDL in the arterial subendothelial layer provokes monocyte generation from progenitor cells in the BM and their subsequent release into the circulation. Under the influence of chemokines like MCP-1, circulating monocytes migrate to inflammatory atherosclerotic lesions/plaques, where they differentiate into DCs or macrophages following infiltration through the endothelium. Inflammatory macrophages release chemokines/cytokines, which promote plaque inflammation. Macrophages initially help to reduce LDL load, however, upon the excessive sink of oxLDL they convert into foam cells that further release cytokines/chemokines, thereby escalating inflammation or being deposited in the plaque, resulting in plaque growth. As the plaque grows, it becomes unstable and may rupture. Due to the inflammatory milieu of the plaque, procoagulant factors are activated and fibrin production increases. Collectively, these changes result in blood coagulation at the rupture site and the formation of thrombus. In addition, foam cells necrosis and other inflammatory cells build up the necrotic core. While efferocytosis prevents inflammation and plaque growth, the impaired efferocytosis of apoptotic/necrotic bodies may lead to further deposition of macrophages/foam cells in the plaque. Due to the inflammatory conditions of the plaque, monocytes differentiate into DCs, which infiltrate the endothelium and release proinflammatory cytokines that promote inflammation and atherosclerosis. Similarly, due to the stimulation by inflammatory mediators, neutrophils are differentiated and infiltrate through the endothelium to the plaque, where they release proinflammatory cytokines that promote inflammation and atherosclerosis. Neutrophil-released NETs also precipitate endothelial damage and cell death, which may lead to plaque rupture.

Cholesterol is the primary lipid component accumulated in both free and esterified forms in atherosclerotic plaques, typically as the low-density lipoprotein (LDL) [16, 17]. Hence, atherosclerosis is classified as a cholesterol storage disease caused by the retention of LDL in arteries intima. After accumulation in the arterial wall, LDL undergoes modifications before being taken up by macrophages via **scavenger receptors**, a process known as phagocytosis, and other mechanisms, ultimately leading to its accumulation in macrophages and driving the plaque formation (Fig. 1) [18–21]. Consequently, the increased levels of cholesterol and LDL in the plasma are closely linked to the development of atherosclerosis [22, 23]. Indeed, LDL is the predominant carrier of cholesterol in the plasma, delivering cholesterol to the liver and other tissues [24]. Mechanistically, the LDL receptor (LDLR) in hepatocytes recognizes and binds to ApoB-100 (apolipoprotein B-100) and Apo-E (apolipoprotein E) on LDL, thus mediating LDL uptake [24]. This process is referred to as endocytosis [24]. Therefore, genetic defects or the ablation of *LDLR* (*Ldlr*^−/−^) or *APOE* (*Apoe*^*−/−*^) in mice leads to atherosclerosis by increasing LDL levels in the blood [25].

One of the key events in atherogenesis is the cumulative oxidation of aggregated LDL within the plaque [26, 27]. The oxidation of LDL promotes its uptake by macrophages in the intimal layer [28]. Although macrophages can uptake LDL via **micropinocytosis**, aggregated oxidized LDL (oxLDL) and cholesterol crystals are primarily taken up by phagocytosis. As a result, the excessive influx of oxLDL and other ApoB-containing lipoproteins leads to the formation of intracellular lipid droplets thus turning macrophages into cholesterol-laden cells, termed “foam cells”. Foam cells release proinflammatory cytokines that induce the recruitment of myeloid cells and the ignition of inflammatory immune responses within the plaque (Fig. 1) [29]. Hence, beyond the primary events, inflammation plays a decisive role in the exacerbation of the plaque and the progression of atherosclerosis [29].

Stable plaques are featured by chronic low-grade inflammation, while unstable plaques exhibit active inflammation, which further promotes plaque rupture and vulnerability by thinning the fibrous cap [29]. Clinically, macrophage infiltration, M1 polarization, and the expression of anti-/pro-inflammatory cytokines were elevated in epicardial fat tissue of coronary artery disease patients, suggesting a link between coronary atherosclerosis and inflammatory macrophages [30]. These findings corroborate the indispensable role of inflammation in the progression of atherosclerosis.

In this review, we aimed to extensively divulge molecular and cellular mechanisms of inflammation in atherosclerosis based on recently identified cutting-edge findings. Our effort may lay out potential molecular targets and signaling pathways with therapeutic interests in atherosclerosis. Of note, given the extensive body of literature, referring to other prominent reviews (e.g. [31–37],) is highly advisable.

### The implication of macrophages in atherosclerotic Inflammation

Monocytes are a type of leukocytes (white blood cells) that originate from the bone marrow (BM) then circulate in the blood and finally migrate to different body tissues, where they are converted into macrophages [38]. Subsequently, based on the localized milieu and stimuli, macrophages differentiate into two primary subtypes; proinflammatory M1 macrophages and anti-inflammatory M2 macrophages [39]. Typically, lipopolysaccharides (LPS) (derived from the outer membrane of gram-negative bacteria) and IFN-γ cytokine (mainly produced by T cells and natural killer cells) induce the polarization of M1 macrophages, while IL-4 and IL-13 cytokines (primarily produced by T helper 2 cells) trigger the polarization of M2 macrophages [40, 41]. Macrophage polarization constitutes alterations in signaling pathways, transcription, and post-transcription networks and is regulated by effectors like glucocorticoids (steroid hormones involved in metabolism), immunometabolism, and microRNAs [42, 43]. It appears that dramatic shifts in cellular metabolism may regulate macrophage polarization [44, 45]. Mounting evidence suggests that aerobic glycolysis is more pronounced in M1 macrophages, while M2 macrophages more likely display mitochondrial oxidative phosphorylation [46, 47]. Glycolysis produces less ATP but is activated more rapidly than mitochondrial respiration thus enabling an agile immune response in M1 macrophages [46–48]. Mechanistically, in M1 macrophages (activated by LPS), *SLC2A1*/GLUT-1 gene expression increases, leading to enhanced glucose uptake and glycolysis [49]. Increased glycolysis also contributes to the M1 polarization of macrophages. In one mechanism, LPS promotes macrophage gene expression and the protein levels of *PKM*/PKM2, a glycolytic enzyme that interacts with HIF-1α transcription factor to promote its activation. Subsequently, HIF-1a transactivates glycolytic and pro-inflammatory genes, thereby prompting glycolysis, conferring pro-inflammatory phenotype, and driving M1 polarization [50]. Besides this brief illustration, a recent review has attempted to illuminate on the role of immunometabolism in macrophage polarization in more detail [43].

Histological studies have demonstrated that M1 macrophages are predominant in atherosclerotic plaques and laden with LDL, whereas M2 macrophages are less commonly found in plaque sites and contain less fat (Box 1) [51]. Also, a distinct subtype of macrophages, termed MOX cells, has been isolated from mouse aortic plaques. Their phenotypes are largely driven by oxLDL or ox-phospholipids and therefore differ from the M1 and M2 subtypes [52]. Thus, macrophages found in atherosclerotic plaques are plastic and heterogeneous. We hypothesize that MOX cells were indeed M1 macrophages that may be differentiated into a more pathological form through largely unidentified mechanisms but were likely turned on by oxLDL or its excessive oxLDL within these cells. Therefore, here, the term “macrophages” refers to M1 macrophages or those phenotypes that are predominantly involved in atherosclerosis or present in atherosclerotic plaques.

A high level of circulating cholesterol or LDL (**hypercholesterolemia**) induces the proliferation of hematopoietic stem or progenitor cells in the BM, thereby increasing circulating monocytes (systemic monocytosis) and their inflow into arterial subendothelial walls and plaque sites [53, 54]. In addition to hypercholesterolemia, chemotactic cytokines (chemokines) can further increase monocytes recruitment to the plaque; for instance, *CCL2*/MCP-1 cytokine can bind to CCR2 receptor on **classical monocytes** and induce their migration to the plaque (Fig. 1) [55]. Subsequently, the accumulated monocytes, under the influence of activating stimuli present in plaque sites such as cytokines differentiate into macrophages that excessively uptake LDL and transform into foam cells [56].

Initially, plaque-resident macrophages display anti-inflammatory roles before turning into foam cells likely due to enrichment with anti-inflammatory genes (Fig. 1) [57, 58]. However, the excessive uptake of oxLDL not only converts these macrophages into foam cells but also exerts changes on intracellular signaling pathways in a manner that favors proinflammatory responses, thereby conferring proinflammatory traits on foam cells as the plaque advances [59]. Yet, the mechanisms and nature of these changes are not fully understood. In a recent study on human carotid endarterectomies in different cohorts, using cutting-edge techniques (e.g., single-cell RNA sequencing, bulk RNA sequencing, and immunohistochemistry) revealed a unique subset of macrophages with a high expression of two specific genes (*PLIN2*^hi^/*TREM1*^hi^), conferring these cells enhanced lipid uptake in association with proinflammatory traits [60]. This study supports the notion that enhanced lipid uptake is associated with the proinflammatory traits of macrophages in atherosclerotic plaques.

Some argue that oxLDL is not the only factor that contributes to the proinflammatory features of foam cells. Indeed, other events occurring at the plaque site or within the arterial wall, such as the accumulation of cellular debris (apoptotic and necrotic bodies), the presence of hypoxia, and hyperglycemia (as observed in diabetic patients) also trigger the activation of certain signaling pathways in foam cells. These pathways rewire foam cell metabolism and induce epigenetic modifications, leading to the activation of proinflammatory genes in the later stages of atherosclerosis [59]. Therefore, oxLDL, hypoxia, hyperglycemia, and apoptotic/necrotic debris are among the potential factors that likely induce the shift from initial anti-inflammatory responses of macrophages to foam cells’ proinflammatory traits during the advanced stages of atherosclerosis (Fig. 1). This is the direction in which research should proceed to uncover additional factors in atherosclerotic milieu that may contribute to this shift and the associated mechanisms. A complete understanding of these mechanisms may enable us to effectively halt the inflammation driven by foam cells and the progression of atherosclerosis.

Foam cells release proinflammatory cytokines and chemokines that provoke a proinflammatory response and the recruitment of other immune cells to the plaque (Box 2), respectively, leading to the escalation of inflammation and the progression of atherosclerosis. Ultimately, excessive lipid accumulation within foam cells may induce **necrosis** or apoptosis, resulting in the formation and deposition of cellular debris as apoptotic and necrotic bodies (Fig. 1) [61]. Potential mechanisms have been proposed for the oxLDL-mediated induction of cell death in foam cells. In one mechanism, following oxLDL uptake, lipid peroxides modify cellular proteins and lipids, trigger oxidative stress, and modulate numerous signaling pathways and the expression of diverse genes. Together, these changes result in an overload of Ca^2+^ in the cytosol, which triggers apoptosis or necrosis [62]. Besides, an increased level of Ca^2+^ in the cytosol initiates its entry into the mitochondria and the nucleus, leading to mitochondrial apoptosis and nuclear events such as gene expression modulations that promote apoptosis [63]. However, oxLDL-mediated cell death mechanisms in foam cells are complex and beyond the scope of this review. Further literature reviews may represent some of these mechanisms but additional research is necessary to fully understand them.

Ample researchers contend that cellular debris may drive further formation of foam cells from macrophages thus creating a vicious cycle that amplifies the number of foam cells and exacerbates inflammation (Fig. 1) [59]. However, the remnants of foam cells can be cleared by neighboring non-foamy macrophages through a process called **efferocytosis** (Fig. 1) [61]. These macrophages are termed efferocytes and prevent inflammation, however, their function is rather impaired during atherosclerosis [61]. For instance, a recent study reported that during atherosclerosis, macrophage CD147 impedes efferocytosis by inhibiting GAS6, which is a bridging molecule that facilitates macrophage attachment for encapturing apoptotic bodies [64, 65]. Consequently, in mice, the genetic deletion of *Cd147* or its inhibition by a monoclonal antibody prevents inflammation and atherosclerosis by enhancing efferocytosis [64]. Another study revealed that during atherosclerosis, oxLDL mediates the upregulation of CD147 by activating the PI3K-Akt-mTOR signaling pathway in macrophages [66]. Based on these findings, we postulate that it is indeed oxLDL or its excessive uptake that primarily causes the impairment of efferocytosis during atherosclerosis. Nonetheless, future studies are warranted to uncover additional mechanisms.

Box 1 Promoting M2 polarization may serve as an atheroprotective strategy Anti-inflammatory macrophages, likely categorized as M2 macrophages, are found in the early stage of atherosclerosis and have been shown to elicit atheroprotective and anti-inflammatory effects [140]. However, in the late stages, the M2 population is predominantly replaced by or converted to M1 macrophages [141]. It is hypothesized that increased M2 polarization may modulate or reduce the population of M1 macrophages in plaques. Supporting this hypothesis, the exposure of *Apoe*^*−/−*^ mice to ^137^Cs (γ) radiation (low to moderate doses) significantly skewed BM-derived macrophages toward the M2 phenotype on the first day of exposure [142]. Also, IL-1β secretion from these cells decreased, while IL-10 secretion increased, also supporting M2 polarization [142]. Under long-term exposure (100 days), Ly6C^Low^ M2 monocytes were markedly increased in the spleen. Furthermore, the plaque content of CD68^+^ cells (mainly upregulated in M1 macrophages) was significantly reduced as the exposure dose increased [142]. These findings suggest that ionizing radiation therapy promotes the M2 polarization of circulating and spleen macrophages and reduces M1 macrophage content in the plaque, thereby favoring anti-inflammatory and atheroprotective responses.

Besides ionizing radiation, mesenchymal stem cells (MSCs) could also be used for promoting M2 polarization. In an independent study, the administration of MSCs-derived exosomes to *Apoe*^*−/−*^ mice enhanced M2 polarization and reduced macrophage infiltration and plaque size [143]. To identify the underpinning mechanism, mouse RAW264.7 macrophages were incubated with these exosomes in vitro [143]. Exosomes were found to transfer *miR-21a-5p* to macrophages, which suppressed *KLF6* mRNA, leading to M2 polarization [143]. Additionally, *miR-21a-5p* inhibited *MAPK1*/ERK2 mRNA, thereby inhibiting macrophage migration [143].

Altogether, these findings suggest that increasing the M2 polarization of the macrophage population in the circulation or spleen through methods such as radiation therapy or MSCs-derived exosomes may reduce M1 macrophage content in plaques. This reduction is associated with decreased plaque size and inflammation, thereby offering a promising therapeutic strategy.

Box 2 Role of dendritic cells and neutrophils in atherosclerosis Dendritic cells (DCs) are innate immune cells involved in atherosclerosis by directly uptaking lipids, mediating efferocytosis, and presenting plaque antigens to T cells, casing their activation and recruitment to plaque sites. DCs also release multiple cytokines and chemokines that mediate the plaque mobilization of other immune cells such as monocytes/macrophages [144]. The BATF3-dependent CD8α^+^ subset of DCs expresses *BATF3*/BATF3 transcription factor [145]. In *Apoe*^−/−^ mice, *Batf3* deletion (*Batf3*^−/−^) attenuated CD8α^+^ DCs, thereby reducing the localization of macrophages to aortic plaques, resulting in the suppression of inflammation and the prevention of atherosclerosis [145]. Hence, CD8α^+^ DCs play an atherogenic role by increasing the recruitment of macrophages to plaques thus promoting inflammation. For that purpose, CD8α^+^ DCs release IL-12 cytokine, which induces the proliferation and differentiation of T helper 1 (Th1) cells. In turn, Th1 cells immensely produce and release IFN-γ, which binds to its receptor on macrophages thus upregulating the expression and secretion of *CCL5*/MCP-1 chemokine [145]. This chemokine induces the recruitment and infiltration of other leukocytes and macrophages into plaques, which aggravate atherosclerosis [145]. This profound study demonstrated that the interplay between DCs and Th1 cells drives macrophage response and inflammation in atherosclerotic plaques. In addition, CD8α^+^ DCs specifically express *CLEC9A*/DNGR-1 receptor that recognizes/senses necrotic bodies [146]. In this regard, the whole-body deletion of *Clec9a* in *Apoe*^−/−^ mice or its specific deletion in the BM of *Ldlr*^−/−^ mice significantly attenuated leukocyte content (macrophages and T cells) and increased the expression of *IL10*/IL-10 (anti-inflammatory cytokine) in arterial plaques, thereby diminishing atherosclerosis [146]. *CLEC9A* deletion in CD8α^+^ DCs also promoted *IL10*/IL-10 expression [146]. Therefore, upon *CLEC9A* deletion, the increased expression of *IL10*/IL-10 in the BM and CD8α^+^ DCs seem to be the main cause of reduced recruitment or the activation of macrophages in plaque sites, as well as the alleviation of atherosclerosis [146]. These findings suggest that CD8α^+^ DCs promote inflammation and atherosclerosis mainly through the DNGR-1-mediated downregulation of *IL10*/IL-10, which activates macrophages and increases their plaque load. Collectively, CD8α^+^ DCs play atherogenic roles by inducing the chemotactic recruitment of macrophages to the plaque, resulting in the escalation of inflammation and the progression of atherosclerosis. Despite these findings, immense work is needed to fully uncover the interplay between DCs and macrophages in the context of atherosclerosis. It is also attainable to conduct similar studies on the other subsets of DCs and their potential impacts on macrophages and inflammation during atherosclerosis.

Although rarely present in atherosclerotic plaques, neutrophils are innate immune cells that induce the migration and localization of monocytes/macrophages to plaque sites [147]. Hence, their depletion was shown to lower macrophage content in aortic plaques [147]. Generally, neutrophil granules contain LCN2, LL37, α-defensins, cathepsin G, and azurocidin proteins that are released and somehow are connected with chemotaxis and the induction of monocytes/macrophages recruitment to atherosclerotic plaques [148]. Neutrophil extracellular traps (NETs) are released from neutrophils by rupturing the membrane and represent complex and decondensed DNA fibers combined with granule proteins and histones (Fig. 1) [149]. In *Ldlr*^−/−^ mice, the transcriptomic analysis of plaque macrophages in NETs^+^ areas exposed the heightened activation of inflammasome and proinflammatory phenotypes [150]. As such, reducing plaque NETs using deoxyribonuclease 1 suppressed inflammation and resolved atherosclerosis [150]. However, precise molecular mechanisms accounting for the NETs-mediated activation of proinflammatory response in macrophages are poorly understood, demanding additional explorations.

## The molecular mechanisms of macrophage-mediated inflammation in atherosclerosis

### The role of hypoxia

In both human and animal models, hypoxia is present within atherosclerotic plaques and contributes to the pathogenesis of atherosclerosis. The role of hypoxia in atherosclerosis involves a broad range of mechanisms that have been thoroughly discussed in other seminal reviews [67–69]; however, here, we will focus solely on the effects of hypoxia on macrophages and their proinflammatory features within the plaque. In one primary mechanism, hypoxia activates the HIF-1α transcription factor in plaque macrophages, leading to the transactivation of proinflammatory genes such as cytokines (e.g. MIF) [69–71]. *VEGFA* is one of these upregulated target genes that encode VEGF-A, which is a primary cytokine that mediates angiogenesis in the plaque. In part, angiogenesis may lead to plaque destabilization and rupture, causing thrombosis and intraplaque hemorrhages, ultimately increasing leukocyte infiltration into the plaque, thereby promoting inflammation [72, 73]. Besides, as a multifaceted cytokine, VEGF-A exerts chemotactic effects on other monocytes/macrophages, increasing their recruitment to the plaque, thereby escalating inflammation [74–76].

**Fig. 2:**
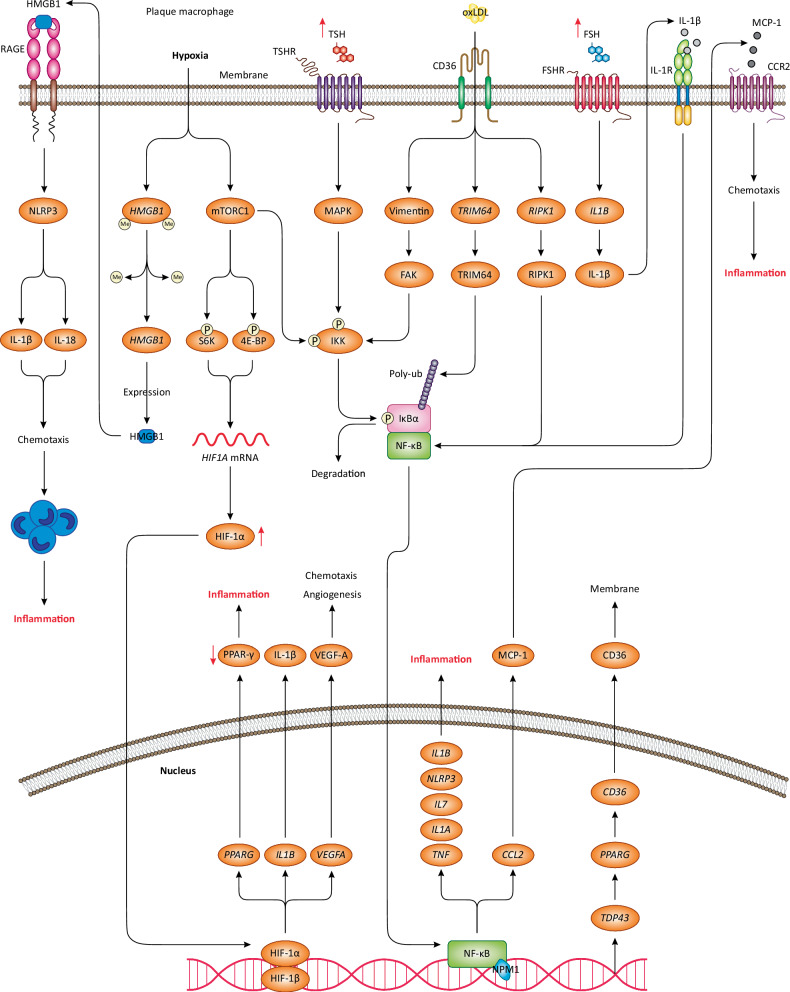
Proinflammatory mechanisms and signaling in atherosclerotic macrophages. Upon plaque hypoxia, two mainstream pathways are activated in macrophages including mTORC1 and HMGB1. The increased transcription and activation of *HMGB1*/HMGB1 stimulate RAGE receptors on macrophage membrane, leading to the activation of NLRP3 inflammasome, which releases IL-1β and IL-18 chemokines, inducing chemotaxis and the recruitment of monocytes/macrophages to plaque sites, thereby promoting inflammation. mTORC1 activation also increases the transcription and expression of *HIF1A*/HIF-1α, which translocates to the nucleus and upregulates the transcription of several genes, playing key roles in angiogenesis and chemotaxis, thereby inducing plaque inflammation. Moreover, mTORC1 phosphorylates and activates the IKK complex, which phosphorylates IκBα, causing its degradation and dissociation from NF-κB, a key transcription factor regulating macrophage inflammation. NF-κB translocates to the nucleus and upregulates the transcription of key proinflammatory genes, cytokines, and chemokines, thus triggering inflammation. *CCL2*/MCP-1 is an key chemokine that can bind to CCR2 receptor on macrophages, inducing chemotaxis and inflammation. The excessively high levels of TSH in the plasma induces MAPK signaling after binding to TSHR receptor on macrophages. Subsequently, MAPK signaling leads to the phosphorylation of the IKK complex, which activates NF-κB. Also, the elevated levels of FSH in the plasma activate FSHR receptor on macrophages, leading to the increased transcription, activation, and release of *IL1B*/IL-1β, which then binds to IL-1R receptor on macrophages, thus amplifiying NF-κB activation. Through unknown mechanisms, *TDP43*/TDP43 is upregulated in macrophages, which upregulates the transcription and activation of *CD36*/CD36 scavenger receptor. CD36 translocates to the membrane and mediates oxLDL/LDL uptake. This results in the activation of vimentin, which induces the phosphorylation of IKK complex by activating FAK kinase, leading to NF-κB activation. In addition, oxLDL uptake is associated with the increased transcription and activation of *TRIM64*/TRIM64 and *RIPK1*/RIPK1 that mediate NF-κB activation. Due to the centralized role of NF-κB in macrophage proinflammatory response, its excessive activation leads to chronic inflammation in the plaque.

CD163^+^ macrophages, found in both mice and human plaques, exemplify cells that are influenced by hypoxia, thereby exhibiting the upregulation of *HIF1A*/HIF-1α and *VEGFA*/VEGF-A, associated with inflammation and plaque growth [77]. Collectively, these data indicate that hypoxia activates the HIF-1α/VEGF-A axis in macrophages, driving plaque inflammation through cytokine release, angiogenesis, and **chemotaxis** (Fig. 2). Moreover, HIF-1α can induce inflammation by downregulating the expression of *PPARG*, which is a key anti-inflammatory gene [78]. This gene encodes anti-inflammatory transcription factor PPAR-γ, which blocks the expression of proinflammatory cytokines and genes (i.e., *IL6*, *IL1B*, *TNF*, *MMP9*, *NOS2*) in macrophages and transactivates anti-inflammatory genes (e.g., *IL10*, *HMOX1*) [79–81]. Hence, we propose that the hypoxia-induced activation of HIF-1α in macrophages may lead to the downregulation of PPAR-γ, thus driving plaque inflammation by reactivating proinflammatory genes and the repression of anti-inflammatory genes (Fig. 2).

Beyond HIF-1α, hypoxia may initiate other signaling pathways in macrophages that exacerbate plaque inflammation. A recent study revealed that chronic intermittent hypoxia leads to the hypomethylation of *HMGB1*, causing its enhanced expression in monocytes/macrophages in a mouse model of atherosclerosis [82]. Subsequently, HMGB1 is released into the extracellular environment and serves as a cytokine that can bind to RAGE receptors and stimulate its signaling in macrophages [82]. This process leads to the activation of the NLRP3 inflammasome, which triggers the activation and release of proinflammatory cytokines including IL-1β and IL-18, thereby promoting the recruitment of monocytes/macrophages to the plaque via chemotactic effects [82]. IL-1β also serves as an autocrine factor, binding to macrophages and initiating intracellular signaling pathways that promote foam cell formation (Box 3) [83]. Collectively, these events escalate plaque inflammation and the progression of atherosclerosis (Fig. 2) [82]. Hence, the hypoxia-mediated activation of HMGB1 in macrophages activates the RAGE-NLRP3 signaling, thus escalating plaque inflammation by promoting chemotaxis and foam cell formation.

mTORC1 is another key factor regulating proinflammatory responses in leukocytes within atherosclerosis context [84]. The plaque hypoxia induces the activation of mTORC1 in macrophages. Subsequently, mTORC1 leads to the phosphorylation of IKK complex components (IKK-α and IKK-β), which activate the IKK complex, leading to the phosphorylation of IκBα (NF-κB inhibitor) and its subsequent ubiquitination and degradation. This liberates the NF-κB transcription factor, allowing it to move to the nucleus and transactivates multiple proinflammatory genes and cytokines (e.g., IL-17, IL-1, TNF-α) (Fig. 2) [67, 85, 86]. Moreover, mTORC1 phosphorylates/activates S6K and 4E-BP that promote translation of *HIF1A* mRNA into HIF-1α, thereby initiating HIF-1α signaling in macrophages (Fig. 2) [87, 88]. Overall, these findings would suggest that hypoxia activates mTORC1 in macrophages, leading to plaque inflammation via NF-κB and HIF-1α signalings.

In addition to the abovementioned mechanisms, hypoxia may modulate more cell signaling pathways and processes in macrophages although their involvement in the exacerbation of plaque inflammation and atherosclerosis remains elusive at this time. This complexity arises from the profound and multifaceted impact of hypoxia on cellular signaling and overall macrophage biology. Besides, plaque macrophages exist within a pathological environment, making the involvement of hypoxia much more complex than in macrophages under normal conditions. As such, the clinical and therapeutic translation of these findings is limited (Box 3). With the current state of knowledge, few options are available to effectively intervene or prevent hypoxia-induced inflammatory mechanisms in macrophages within atherosclerotic plaques.

Box 3 Seminal clinical trials and FDA-approved anti-inflammatory drugs In the main text, we indicated the limitations of targeting inflammation in atherosclerosis. As described earlier, the activation and release of IL-1β by plaque macrophages are associated with chemotactic effects and increased foam cell formation, significantly promoting plaque inflammation and atherosclerosis [82, 83, 151]. Additionally, other plaque cells such as SMCs, ECs, and immune cells also activate and release IL-1β [152]. These facts, along with extensive evidence from animal studies [152], demonstrate the atherogenic role of IL-1β. Hence, IL-1β may serve as a potential therapeutic target in atherosclerosis. Canakinumab is an FDA-approved monoclonal antibody that specifically targets IL-1β [153]. The CANTOS (canakinumab anti-inflammatory thrombosis outcome study) was a double-blind randomized clinical trial that examined the therapeutic efficacy and anti-inflammatory effects of canakinumab in atherosclerosis and its complications [154]. The CANTOS trial included 10061 stable coronary artery disease patients with hs-CRP levels >2 mg/L who were under optimal medical treatment. These patients were randomly assigned to receive either a placebo or canakinumab [154]. Canakinumab was administered subcutaneously to patient groups at different doses (50, 150, 300 mg) with a follow-up of 3.7 years [154]. It was shown that hs-CRP was reduced by 26%, 37%, and 41% in groups receiving 50 mg, 150 mg, and 300 mg of canakinumab, respectively [154]. At 150 mg dosage, canakinumab was an effective anti-inflammatory therapy that significantly reduced recurrent cardiovascular events [154]. The analyses of the CANTOS results also revealed that IL-1β activated downstream IL-6, which was associated with major cardiovascular events [154]. After the first treatment with canakinumab, patients who had the lower levels of IL-6 were less likely to encounter major cardiovascular events, hospitalization, or all-cause mortality [154, 155]. However, canakinumab slightly increased the risk of infection, mortalities, and neutropenia [154]. Therefore, despite these achievements, the CANTOS trial was based on targeting IL-1β, which appears to be a relatively unsafe target due to its crucial role in host defense against infections. This suggests that we have not yet found an ideal target to block inflammation and its complications in atherosclerosis. Hence, additional basic studies are required to identify more favorable targets before conducting further clinical trials on conventional targets.

Colchicine is an FDA-approved anti-inflammatory drug used to treat various diseases such as acute gout, pericarditis, and Behçet disease [152]. For centuries, this alkaloid has been extracted from *Colchicum autumnale* and used for the treatment of joint swelling. The primary anti-inflammatory mechanism of colchicine involves binding to tubulin dimers and inhibiting the polymerization of microtubules within neutrophils and monocytes [156]. This disrupts the cytoskeleton, thereby inhibiting the invasion of neutrophils and monocytes as well as their intracellular trafficking, cytokine release, and inflammation [152, 157]. Recently, a double-blind randomized clinical trial COLCOT (colchicine cardiovascular outcomes trial) was conducted to evaluate the daily intake of low-dose colchicine (0.5 mg) in 4745 patients who experienced myocardial infarction 30 days before the study with a follow-up of 1.8 years [158]. Colchicine treatment significantly reduced ischemic cardiovascular events such as cardiovascular death, angina hospitalization, resuscitated cardiac arrest, and stroke by 23% [158]. However, adverse effects such as abdominal discomfort and nausea were observed in the treatment group [158]. Moreover, chronic colchicine treatment increased pneumonia frequency from 0.4% to 0.9%. Although no infection-associated deaths were reported, the rate of septic shock was as low as the placebo group [158]. Despite the promising outcome of the COLCOT trial, it suffers from nonspecific targeting as colchicine does not act specifically on immune cells or molecular targets and generally suppresses neutrophils and macrophages and their cytokine secretion. This further demonstrates the need to identify specific inflammatory targets to develop more precise anti-inflammatory drugs.

### The role of oxLDL

As described above, the proinflammatory features of macrophages in atherosclerosis are largely attributed to the interaction with oxLDL or its uptake. A recent study on *Apoe*^−/−^ mice revealed that oxLDL triggered the upregulation of *TRIM64* expression, the activation of NF-κB signaling and NLRP3 inflammasome in macrophages, ultimately resulting in plaque inflammation and the development of atherosclerosis [89]. TRIM64 is a type of RING E3 ubiquitin ligase, which was shown to interact with IκBα and mediate its ubiquitination, thereby liberating NF-κB [89]. Then, activated NF-κB translocates to the nucleus and binds to the promoter region of *TRIM64*, upregulating its transcription thus forming a positive feedback loop that amplifies *TRIM64*/TRIM64 expression and NF-κB activation [89]. In addition, NF-κB upregulates *NLRP3* transcription, leading to the activation and priming of NLRP3 inflammasome, which then activates and releases IL-18 and IL-1β cytokines [89]. Hence, oxLDL induces the activation of TRIM64/NF-κB/NLRP3 signaling in macrophages, thereby releasing proinflammatory cytokines, which promote plaque inflammation and atherosclerosis (Fig. 2).

Vimentin is a type III intermediate filament, which is secreted into the extracellular milieu by macrophages in response to proinflammatory stimuli such as cytokines and chemokines (e.g., TNF-α and MCP-1) [90]. An in vitro experiment revealed that the binding of oxLDL to CD36 scavenger receptors on macrophages increases vimentin release up to 7 folds [91]. Extracellular vimentin induces NF-κB signaling in macrophages, leading to the increased transcription and secretion of proinflammatory cytokines such as TNF-α [91]. Mechanistically, extracellular vimentin triggers the activation and phosphorylation of FAK, a kinase protein at focal adhesion sites, which phosphorylates and activates the IKK complex, leading to the prolonged activation of NF-κB (Fig. 2) [91]. It was shown that extracellular vimentin and oxLDL have synergistic effects on NF-κB activation and the release of TNF-α and IL-6 cytokines from macrophages [91]. In line with this, the circulating levels of vimentin were increased 1.5 folds in atherosclerotic patients with coronary artery disease compared with normal individuals [91]. Collectively, the binding and uptake of oxLDL by CD36 induce the activation of vimentin/FAK/NF-κB axis in macrophages, resulting in cytokine release and inflammation.

RIPK1 is an intracellular kinase protein that regulates NF-κB activation in macrophages [92]. In early atherosclerotic lesions in mouse and human models, *RIPK1*/RIPK1 is abundantly expressed in macrophages, leading to plaque inflammation due to the activation of NF-κB signaling and the release of IL-1α and IL-1β cytokines [93]. In vitro, treating M1 macrophages with oxLDL upregulated *RIPK1* expression by 1.8 folds [93]. In mouse peritoneal macrophages, *RIPK1* silencing using antisense oligonucleotides and then treating them with oxLDL reduced inflammatory transcripts such as *NFKB1*, *IL1A*, and *TNF* [93]. In mice, the administration of *RIPK1* antisense oligonucleotides reduced lesion areas and the plasma levels of proinflammatory cytokines and significantly suppressed plaque inflammation and growth [92, 93]. Moreover, using antisense oligonucleotides effectively downregulated *RIPK1* expression without toxicity, while avoiding its complete loss and preventing inflammatory cell death [93]. These findings suggest that in early atherosclerosis, oxLDL induces the activation and transcription of *RIPK1*/RIPK1 in macrophages and subsequently NF-κB signaling (Fig. 2). However, this study did not explore the underlying mechanisms of *RIPK1* expression upon oxLDL exposure or NF-κB activation induced by RIPK1. Therefore, a closer examination of these mechanisms is required.

In summary, the described studies have shed light on the potential underlying mechanisms of macrophage-mediated inflammation after the exposure or uptake of oxLDL in atherosclerotic plaques. However, extensive efforts are still needed to further elaborate on these mechanisms and address the remaining gaps. For instance, it is still unclear how oxLDL increases the transcription and activation of *TRIM64* and *RIPK1* or promotes the secretion of vimentin from macrophages. Indeed, recent research on the impacts of oxLDL on macrophages in the context of atherosclerosis has not been adequately explored. It appears that oxLDL-mediated effects on macrophages may involve several mechanisms and our current understanding of these mechanisms represents only a small piece of the puzzle. Thus, this incomplete knowledge leaves no room for effective therapeutic interventions.

### The binding partners of NF-κB and gene transcription

In atherosclerosis, given the indispensable role of NF-κB signaling in macrophage proinflammatory response, it is critical to reveal how NF-κB can land on its target proinflammatory genes and mediate their transcription. NPM1 is a nucleus-cytoplasmic shuttling protein that mediates NF-kB DNA binding in macrophages and other cell types [94, 95]. Mechanistically, NPM1 facilitates the recruitment and binding of p65 NF-κB subunit to its target genes by enhancing its DNA-binding capacity [95]. In detail, NPM1 serves as a chaperon and interacts with the p65 subunit to enhance its nuclear translocation and localization on target genes, thereby successfully transcribing inflammatory genes, resulting in inflammation in carotid plaques [96]. Thus, the genetic ablation of *NPM1* in human endothelial cells (ECs) was shown to resolve plaque inflammation [96], and similar results may occur in macrophages as well. However, apparently, NPM1 does not function specifically to guide the binding of NF-κB only to inflammatory genes but rather functions generally on all target genes. Since NF-κB regulates the transcription of hundreds of genes, many of which play vital roles in the survival and biology of macrophages, it is crucial to identify specific binding partners and molecular chaperones that selectively regulate NF-κB binding to inflammatory genes. This targeted approach is essential for the effective manipulation of NF-κB signaling and the prevention of inflammation in atherosclerotic macrophages.

### Molecules mediating LDL uptake by macrophages

As discussed earlier, atherosclerotic macrophages have enormous ability to uptake LDL/oxLDL. This raises interest in another intriguing research domain on atherosclerotic macrophages. Increased LDL uptake by macrophages is closely linked to their proinflammatory features during atherosclerosis. For instance, TDP43 is a transcription factor whose upregulation in murine plaque macrophages promotes lipid uptake and foam cell formation [97]. TDP43 mediates these effects by activating another transcription factor, *PPARG*/PPAR-γ, via mechanisms that are not fully delineated. However, it is suggested that TDP43 may directly transactivate *PPARG*/PPAR-γ or modulate other genes involved in the regulation of PPAR-γ activity such as *CTNNB1*/β-catenin or *GSK3B*/GSK3β molecules [98, 99]. Subsequently, PPAR-γ binds to *CD36* gene and promotes its transcription and protein levels in macrophages (Fig. 2) [100, 101]. CD36 is a macrophage scavenger receptor that can bind and uptake oxLDL, leading to foam cell formation and the exacerbation of atherosclerosis [100, 101]. Therefore, the activation of the TDP43/PPAR-γ/CD36 axis is a new mechanistic model that explains increased LDL/oxLDL uptake by macrophages during atherosclerosis. Yet, it needs to be determined what causes TDP43 upregulation in plaque macrophages. Is it hypoxia? Or is it the impact of oxLDL? Or perhaps other events taking place in the atherosclerotic milieu cause *TDP43* transactivation in macrophages! These questions need to be answered and even more studies are required to delineate other mechanisms that could explain the enigmatic ability of plaque macrophages in LDL/oxLDL uptake compared with normal macrophages (Box 4).

Box 4 The role of hormones in macrophage-mediated inflammation in atherosclerosis Hypothyroidism is a condition in which the production and secretion of thyroid hormones from the thyroid gland are reduced, resulting in a compensatory elevation of TSH hormone. TSH is a pituitary gland hormone whose circulating level is independently associated with the prevalence of carotid plaques and intima-media thickness in a cohort of 1103 individuals [159]. This in large is attributed to the role of TSH in regulating macrophage-mediated inflammation [160]. In one mechanism, TSH binds to its receptor TSHR on macrophages, leading to the activation of MAPK signaling, which induces the phosphorylation and activation of the IKK complex, thereby activating NF-κB. Subsequently, NF-κB translocates to the nucleus and upregulates the transcription of proinflammatory genes (e.g., cytokines and chemokines), which promote plaque inflammation and atherosclerosis (Fig. 2) [159]. Hence, increased TSH levels in the plasma activate the MAPK-NF-κB signaling in macrophages, leading to cytokines and chemokines release, monocytes recruitment, and plaque inflammation. Thus, in *Apoe*^*−/−*^ mice, *Tshr* knockout reduced inflammation and macrophage content in atherosclerotic plaques [159]. These findings suggest that the exaggerated activation of NF-κB in macrophages significantly drives plaque inflammation during atherosclerosis. Hence, NF-κB appears to be a potential therapeutic target in macrophages that could prevent plaque inflammation and atherosclerosis. However, some data challenges this hypothesis. For instance, in mice, limiting NF-κB activity unexpectedly increases plaque size and promotes atherosclerosis [161]. Additionally, in mouse macrophages, genetic ablation of *Ikbkb*, which encodes IKK-β (a component of the IKK complex), promotes atherosclerotic lesions and macrophage number in plaques [161]. A possible explanation for these observations is that NF-κB regulates the expression of hundreds of diverse genes, essential for the survival, proliferation, and differentiation of macrophages. Therefore, permanent inhibition of NF-κB is not recommended, as it may interfere with critical biological processes and macrophage function thus adversely affecting atherosclerosis. As such, under the high levels of TSH in the plasma, inhibiting TSHR on macrophages (as suggested by the early study [159]) might be a more favorable strategy for suppressing plaque inflammation than targeting NF-κB.

FSH is another pituitary hormone and its excessively high levels in the blood are linked to atherosclerosis. For instance, menopausal women and prostate cancer patients under androgen deprivation therapy exhibit the abnormally high levels of FSH and therefore are at a high risk of developing atherosclerosis [151]. To determine the underpinning mechanisms, a recent study on *Apoe*^*−/−*^ mice demonstrated that short- and long-term exposure to FSH significantly promoted atherosclerosis, aorta plaques, macrophage content, and plaque instability [151]. On the mechanistic ground, it was found that FSH binds to its receptor (FSHR) on macrophages, leading to the expression of *IL1B*/IL-1β, which exerts chemotactic effects on macrophages thus promoting their infiltration to the plaque and enhancing inflammation [151]. To elicit chemotaxis, the released IL-1β binds to its receptor on monocytes/macrophages, causing NF-κB activation and nuclear translocation to transactivate *CCL2*/MCP-1 chemokine (Fig. 2) [151]. Subsequently, MCP-1 is released and binds to its receptor, CCR2, on monocytes/macrophages thus inducing their activation and chemotactic recruitment [151]. These findings indicate that increased circulating FSH promotes plaque inflammation through macrophage chemotaxis. Taken together, the activation of NF-κB signaling in macrophages and their chemotactic recruitment and localization to the plaque account for the TSH- and FSH-mediated plaque inflammation in atherosclerosis.

## The anti-inflammatory mechanisms of macrophages in atherosclerosis

Besides proinflammatory roles, pre-foam cell macrophages tend to be anti-inflammatory, aiming to resolve inflammation and prevent plaque growth in the early stage of atherosclerosis (Box 1). It is even perceived that oxLDL/LDL uptake by macrophages is initially a protective mechanism, which prevents the formation of lipid deposits in vascular walls in early atherosclerosis. However, as plaque growth continues and LDL uptake exceeds macrophage capacity, which causes LDL retention, macrophages are switched to pathological and proinflammatory phenotypes. Hence, in an optimistic view, discussing anti-inflammatory mechanisms activated in macrophages during the early stage of atherosclerosis may enable us to exploit these mechanisms for inhibiting inflammation and plaque growth in advanced stages. In below, we will discuss recently identified mechanisms in macrophages that seems rather to be anti-inflammatoy and atheroprotective.

### The role of TGR5

**Fig. 3:**
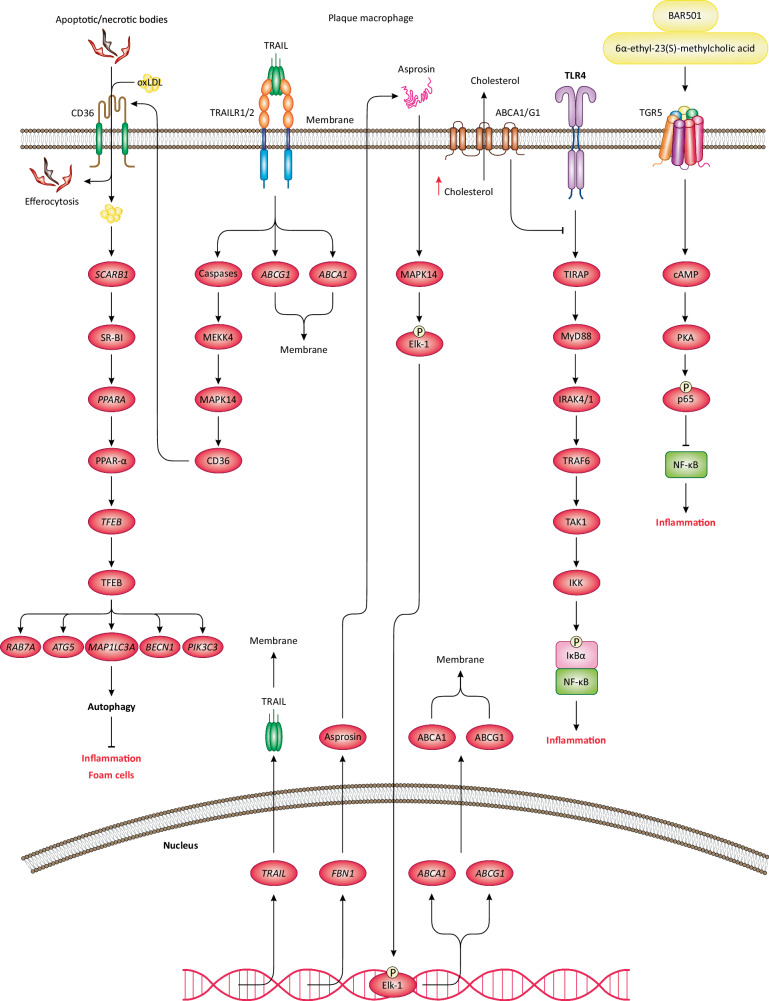
Anti-inflammatory mechanisms and signaling in atherosclerotic macrophages. The increased transcription and protein levels of *TRAIL*/TRAIL in macrophages induce anti-inflammatory response in macrophages. TRAIL cytokine is released and then binds to TRAILR1/2 receptors on macrophages, inducing the activation of caspases/MEKK4/MAPK14 signaling, ultimately, leading to the upregulation of CD36 scavenger receptor. Subsequently, CD36 translocates to the macrophage membrane and mediates efferocytosis or LDL/oxLDL uptake. Also, the activation of CD36 induces the transcription and activation of *SCARB1*/SCARB1 and *PPARA*/PPARA transcription factors, which activate another transcription factor TFEB, thereby promoting the transcription of autophagy genes. Autophagy serves as an anti-inflammatory mechanism owing to its role in reducing LDL/cholesterol load in macrophages and preventing foam cell formation. TRAILR1/2 receptor signaling also upregulates the transcription and protein levels of *ABCA1*/ABCA1 and *ABCG1*/ABCG1 transporters, which translocate to macrophage membrane and mediate cholesterol efflux, thus attenuating lipid load and foam cell formation. Also, the activation of ABCA1/G1 transporters inhibits TLR4 signaling and NF-κB activation by reducing cholesterol content in lipid rafts, thereby undermining TLR4 distribution on macrophage membrane. Moreover, inducing the transcription and upregulation of *FBN1*/asprosin in macrophages activates MAPK14 signaling and Elk-1 transcription factor, which translocates to the nucleus and upregulates the transcription of *ABCA1* and *ABCG1* genes, thereby promoting the reverse cholesterol efflux, ultimately, preventing foam cell formation and inflammation. Last but not least, inducing TGR5 activation on macrophages leads to cAMP generation in the cytosol, which activates PKA. In turn, PKA phosphorylates the p65 NF-κB subunit, resulting in NF-κB inhibition, which retards inflammation.

TGR5 is a G protein-coupled bile acid receptor that is highly expressed in macrophages [102]. In mice intraplaque macrophages, TGR5 activation leads to cAMP production in the cytosol, which activates PKA to phosphorylate p65 NF-κB subunit in C-terminal DNA-binding domain [103–105]. As a result, NF-κB is inhibited, leading to the repression of proinflammatory genes thus reducing cytokine release and inflammation (Fig. 3) [103–105]. As such, the activation of the TGR5/PKA/NF-κB axis in macrophages is a protective anti-inflammatory mechanism that prevents plaque inflammation and atherosclerosis. To activate TGR5 on macrophages, 6α-ethyl-23(S)-methylcholic acid, a selective agonist of TGR5 was used [103]. Given that the TGR5/PKA/NF-κB axis is not typically activated in plaque macrophages, TGR5 agonists are required for its activation and enjoying its atheroprotective merits. A recent study demonstrated that using BAR501, another selective TGR5 agonist, alleviated aorta thickness, macrophage content, and atherosclerotic lesions in *Apoe*^−/−^ mice due to TGR5 activation [106]. Moreover, modulating the intermediate components of TGR5 signaling could be an alternative approach to activate TGR5 and inhibit NF-κB in macrophages.

### The role of TRAIL

TRAIL is a cytokine from TNF superfamily, which contributes to M1 macrophage polarization by activating NF-κB and the transcription of proinflammatory genes and cytokines [107, 108]. In turn, NF-κB upregulates *TNFSF10*/TRAIL transcription thus creating a positive feedback loop that amplifies TRAIL expression and NF-κB activation, which further promotes inflammation [109, 110]. The immune cells of atherosclerotic plaques such as macrophages, T cells, natural killer cells, and DCs can produce and release TRAIL cytokine [111]. Hence, plaque macrophages are likely exposed to TRAIL cytokine, which binds to TRAIL receptors (TRAILR1 and TRAILR2) on macrophages and modulate them in an autocrine manner [111]. In addition to its proinflammatory role, *TRAIL* upregulation in mouse macrophages was closely linked to an enhanced efferocytosis thus averting plaque inflammation and atherosclerosis [110]. In compliance, TRAIL circulating levels and *TRAIL* mRNA levels in monocytes were significantly reduced in coronary artery disease patients. In mice, *Trail*^−/−^ macrophages also promoted atherosclerosis and plaque size, while the reconstitution of *Trail*^+/+^ macrophages alleviated atherosclerosis [110]. Indeed, *Trail*^−/−^ macrophages were proinflammatory and exhibited impaired efferocytosis [110]. From mechanistic viewpoint, TRAIL induces caspase activation and the MEKK4/p38 MAPK (MAPK14) signaling in macrophages, leading to the transcription and upregulation of scavenger receptors such as CD36, thereby improving efferocytosis and LDL uptake (Fig. 3) [112–115]. Moreover, *Trail* ablation in mouse macrophages significantly reduced *ABCA1* and *ABCG1* mRNAs (encoding reverse cholesterol transporters) thus dampening cholesterol efflux and promoting lipid-laden macrophages in plaque sites [110]. Collectively, these findings suggest that *TRAIL*/TRAIL expression in macrophages is rather an anti-inflammatory atheroprotective mechanism mainly due to an increased efferocytosis and reverse cholesterol transport, which respectively prevents inflammation and foam cell formation. Importantly, plaque cytokines differently regulate *TRAIL* expression in macrophages. For instance, TNF-α and IL-18 downregulate *TRAIL* expression, while IFN-γ upregulates it [110, 116]. As such, *TRAIL*/TRAIL upregulation does not typically occur in atherosclerotic macrophages. Therefore, to exploit its therapeutic benefits, strategies must be developed to upregulate its expression in these cells. Further studies are needed to conclusively determine whether the anti-inflammatory benefits of TRAIL in macrophages outweigh its potential proinflammatory effects.

### The role of reverse cholesterol transporters: ABCG1 and ABCA1

Reverse cholesterol transport/efflux is a mechanism that removes excess cholesterol from peripheral tissues and cells, such as macrophages, and transfers it to circulating HDL (good cholesterol). HDL then delivers the cholesterol to the liver for recycling and disposal, thereby preventing plaque buildup and inflammation in arteries [117, 118]. In macrophages, this process is mediated by ATP-binding cassette (ABC) transporters, including ABCG1 and ABCA1, which help prevent foam cell formation [117]. ABCA1 and ABCG1 are localized on the plasma membrane of macrophages and expel free cholesterol to extracellular receptors, primarily HDL, where it is received by the HDL component ApoA1 [117, 119]. This explains the inverse correlation between circulating HDL levels and the development of atherosclerosis observed in multiple clinical and epidemiological studies [120]. The detailed mechanism involves ABCA1 interacting with ApoA1 to transfer cholesterol, producing immature pre-β-HDL, which then transforms into α-HDL. Subsequently, ABCG1 further transfers cholesterol to α-HDL, saturating it to produce mature HDL3 and enlarged HDL2, which is ultimately uptaken by the liver [121, 122]. This mechanism suggests that increasing plasma HDL levels (e.g., by including olive oil in daily nutrition) could significantly reduce the risk of cholesterol-laden macrophages and atherosclerosis. However, this notion requires experimental and clinical corroboration.

In one primary study, the activation of ABCG1 and ABCA1 on macrophages regressed plaque inflammation in atherosclerotic mice, likely due to reverse cholesterol efflux and TLR4 signaling inhibition (TLR4-MyD88, TLR4-TRIF pathways). Indeed, by mediating reverse cholesterol efflux, ABCA1 diminishes cholesterol in lipid rafts, thus restraining the membrane mobilization and distribution of TLR4, thereby reducing TLR4 signaling (Fig. 3) [123]. As such, *ABCA1* ablation in macrophages triggers a proinflammatory response by activating TLR4 signaling [123]. In compliance, in mouse macrophages, a recent study reported that the inducible overexpression of *FBN1*/asprosin (a protein hormone) enhanced reverse cholesterol efflux by upregulating the transcription of *ABCG1* and *ABCA1* [124]. Following its release from macrophages, asprosin can bind to macrophages and induce MAPK14 signaling, leading to the phosphorylation and activation of the Elk-1 transcription factor, which translocates to the nucleus and upregulates *ABCG1* and *ABCA1* [124]. Ultimately, macrophage lipid burden is reduced, lesion areas are shrunk, and plaque stability is improved [124]. However, the anti-atherogenic role of asprosin remains questionable. A recent study demonstrated that asprosin activates proinflammatory TLR4 signaling in macrophages, which may promote plaque inflammation [125]. On the other hand, the activation of the ABC transporters such as ABCA1 and ABCG1 in macrophages may serve as an anti-inflammatory mechanism during atherosclerosis. These transporters could be of therapeutic interest for preventing foam cell formation and resolving inflammation in atherosclerosis, particularly during advanced stages when macrophages are heavily laden with LDL. To modulate the gene expression or activation of these transporters effectively, we need to expand our understanding of the signaling pathways and molecules that regulate ABC transcription in macrophages during atherosclerosis. Furthermore, the success rate of such interventions should be examined in both pre-clinical and clinical settings.

### The role of autophagy

Macroautophagy, commonly referred to as “autophagy”, is an evolutionarily conserved self-eating cellular process, involving the formation of a double membrane vesicle, known as autophagosome, which encaptures its target cargoes such as long-lived organelles and cellular components then fuses with lysosomes for the ultimate degradation and recycling of these cargoes [126–130]. Ample evidence suggests that autophagy activation in macrophages rescues from inflammation and atherosclerosis [131, 132]. In advanced mouse and human coronary plaques, *Lamp2* ablation in macrophages, a key element of chaperone-mediated autophagy (CMA), stimulated NLRP3 inflammasome, leading to IL-1β release and activation, which provoked plaque inflammation [133]. CMA is a specific form of autophagy, typically activated under nutrient deprivation, which involves lysosomal proteolysis and the degradation of cytosolic proteins, mediated by several chaperone molecules [134]. NLRP3 inflammasome is a target protein of CMA, therefore, CMA impairment or *LAMP2* deficiency led to the preservation and excessive activation of NLRP3 inflammasome, which triggered inflammation[133]. Hence, restoring *LAMP2* expression reinitiated CMA in macrophages and prevented plaque inflammation. These findings demonstrate the atheroprotective role of CMA in macrophages. Meanwhile, by eliminating proteins or enzymes involved in lipid metabolism and uptake, CMA can crucially regulate lipid uptake and lipogenesis[135]. Hence, in mice, *LAMP2* deficiency induced the formation of lipid-laden macrophages due to CMA defects[136]. Hence, activating CMA in macrophages can prevent foam cell formation, plaque inflammation, and atherosclerosis. However, we need to determine if we can effectively activate CMA in macrophages of atherosclerotic plaques.

Similar to CMA, autophagy dysfunction may promote foam cell formation[137]. In mice and in vitro models, the macrophage-specific downregulation of *ATG14*/ATG14 expression (a key protein in autophagy) promoted atherosclerosis due to oxLDL accumulation and the induction of inflammation [138]. However, inducing *ATG14* overexpression reactivated autophagy and rescued from plaque inflammation [138]. Therefore, both macrophage autophagy and CMA are atheroprotective and anti-inflammatory. In an independent study, *SCARB1*/SR-BI was found to play a decisive role in autophagy activation in macrophages under lipid engorgement in the aortic plaques of *Apoe*^−/−^ mice [139]. However, *SCARB1* silencing in macrophages increased foam cell formation by 2.5 folds and upregulated the transcription of proinflammatory genes and cytokines induced by oxLDL [139]. Moreover, in *SCARB1*^−/−^ macrophages, autophagy activation by pharmacological means failed to reverse foam cell formation, suggesting that SR-BI is an indispensable factor for autophagy activation in macrophages during LDL uptake [139]. Mechanistically, it was found that SR-BI induced autophagy by upregulating the transcription of *TFEB*/TFEB, a key transcription factor that regulates the expression of key autophagy genes such as *BECN1*/beclin-1, *PIK3C3*/vps34, *RAB7A*/Rab7, *MAP1LC3A*/LC3A, and *ATG5*/ATG5 [139]. In addition, SR-BI triggers the activation and expression of another transcription factor, PPAR-α, through largely unidentified mechanisms. Subsequently, PPAR-α binds to the promoter region of *TFEB*, mediating its transcription thus leading to the expression of key autophagy genes and autophagy activation (Fig. 3) [139]. These findings indicate that autophagy activation in advanced plaque macrophages is a protective response against foam cell formation that functions by reducing LDL load and preventing oxLDL-induced inflammation. Of note, the SR-BI/PPAR-α/TFEB axis is just one mechanism of protective autophagy in macrophages and further investigation may reveal other potential mechanisms. Also, in the case of the SR-BI/PPAR-α/TFEB axis, it remains unclear from a mechanistic standpoint how excessive LDL uptake can modulate or activate the gene expression of *SCARB1*/SR-BI. Furthermore, it is not understood how autophagy activation in macrophages interferes with and reduces LDL load or prevents the initiation of inflammatory signaling driven by oxLDL. Delving into these gaps could enable us to effectively exploit autophagy activation in macrophages for therapeutic purposes, such as preventing foam cell formation and plaque inflammation in atherosclerosis (Box 5).

Box 5 Other concepts of inflammation in atherosclerosis

- ***Hemodynamic disturbances***
  Hemodynamics refers to the patterns of blood flow in the vasculature, which are not uniform throughout the vascular system. While the blood flow is typically laminar in straight parts, it becomes disturbed in arterial branches and curvatures, resulting in irregular, nonuniform, and low shear stress in the vessel walls [162, 163]. These disturbances in the blood flow and altered shear stress play key roles in the formation of atherosclerotic plaques [164]. In one mechanism, these hemodynamic disturbances influence ECs and smooth muscle cells (SMCs), likely through mechanical stress, imparting atherogenic and pro-inflammatory characteristics to these cells [165–167]. In support of this, disturbed blood flow (also known as atheroprone flow) has been shown to upregulate proinflammatory genes (e.g., *VCAM1*, *IL8*, *CCL2*) in cocultured ECs and SMCs, thereby eliciting inflammation [168]. In the aortic ECs of transgenic atherosclerotic mice, atheroprone flow induced *SREBP2* overexpression and its protein levels, which translocated to the nucleus and transactivated NOX2 and NLRP3, leading to the activation of NLRP3 inflammasome [169]. As a result, inflammation increased and in synergy with hyperlipidemia (high-fat diet) exacerbated atherosclerosis [169]. These findings suggest that hemodynamic disturbances enhance atherosclerotic inflammation by modulating ECs or SMCs in the vasculature. However, the investigation of this notion is largely dismissed in recent literature, indicating a need for further studies in this domain.
- ***Inflammation resolution and the role of lipid mediators***
  Both the initiation and resolution of inflammation are parts of the host defense response and the physiology of the body [170]. Thus, if the resolution phase of inflammation is defective, inflammation will persist, leading to a pathological state and chronic diseases such as atherosclerosis [171]. The common features of inflammation resolution include reduced leukocyte recruitment, increased efferocytosis, tissue damage repair, and the suppression of inflammation without immunosuppression [172]. Inflammation resolution is an active process that is highly coordinated by several lipid or non-lipid mediators [172, 173]. Here, we limit our discussion to the lipid mediators of inflammation resolution, for information on non-lipid mediators other prominent reviews can be referred to [171, 174].

Fatty acids participate in inflammation pro-resolving responses in atherosclerosis [175]. For example, in the mouse models of neointimal hyperplasia, omega‐3 polyunsaturated fatty acids were endogenously generated, and their high levels significantly attenuated femoral arterial thrombosis, vascular inflammation, and neointimal hyperplasia in carotid arteries [175]. These events were largely attributed to the interaction between these fatty acids and FFAR4 receptors on vascular cells and macrophages [175]. This interaction prohibited the infiltration of macrophages, thereby significantly improving inflammation resolution under neointimal hyperplasia and thrombosis. Thus, omega‐3 polyunsaturated fatty acids can induce inflammation resolution by activating FFAR4 signaling in the vasculature.

Specialized proresolving mediators (SPMs) are a unique category of fatty acid-derived bioactive lipids [176]. Resolvin D2, an SPM, represses the expression of *IL1B* and attenuates IL-1β secretion in the BM-derived activated macrophages in vitro [177]. Additionally, in peritoneal macrophages, resolvin D2 remarkably reduces the priming/activation of NLRP3 inflammasome and IL-1β secretion both in vitro and in vivo [177]. These effects were found to be mediated by the binding of resolvin D2 to GPR18 receptor [177]. Hence, resolvin D2 mediates inflammation resolution by inducing GPR18 signaling, thus inhibiting the NLRP3 inflammasome and pro-inflammatory cytokines (e.g., IL-1β). Importantly, during an inflammatory response, SPMs are synthesized by various cell types, particularly macrophages and neutrophils, to mediate the resolution phase of inflammation. The big question is that what causes the failure of inflammation resolution during atherosclerosis despite the fact that macrophages and other immune cells can produce SPMs mediators? Does atherosclerosis pathology affect the production and release of SPMs by macrophages? Does the concentration of these mediators determine their efficacy against atherosclerotic inflammation? Can we employ these mediators as anti-inflammatory therapeutic drugs? These questions have been poorly explored by the current literature, thus more in-depth studies are needed. In one study, targeted mass spectrometry was used to identify SPMs in the stable and vulnerable plaques of human carotid atherosclerosis [178]. It was found that resolvin D1 was significantly reduced in the vulnerable plaques compared with the stable ones [178]. SPMs were also depleted in the advanced plaques of high-fat diet *Ldlr*^−/−^ mice [178]. The administration of resolvin D1 restored plaque stability, suppressed plaque necrosis and oxidative stress, and enhanced efferocytosis and fibrous cap thickness [178]. These findings indicate that reduced SPMs levels are associated with the development of unstable plaques due to defective inflammation resolution.

## Conclusion and future perspectives

Arguably, macrophages are the most important players in atherosclerosis and chronic inflammation in plaques. In this review, we focused on the molecular mechanisms and signaling pathways of inflammation, occurring in the macrophages of atherosclerotic lesions/plaques. We delineated how the activation of these mechanisms ignites inflammation, which then transitions to chronic inflammation in plaques, ultimately, exacerbating atherosclerosis. Thus, atherosclerosis can be viewed as a vicious cycle that amplifies inflammation, as most events during its pathophysiology (e.g., the accumulative recruitment/activation of monocytes/macrophages and other immune cells, the presence of hypoxia and oxLDL, increased angiogenesis, etc.) tend to elevate some aspects or modes of inflammation.

Although comprehensively discussing the mechanisms of inflammation in atherosclerosis would require additional literature reviews, we concentrated our discussion on seminal studies in recent years. We focused on key items such as hypoxia, oxLDL and LDL uptake, hormonal changes, transcription factors, cytokines and chemokines, and the crucial role of NF-κB activation in inflammation. Where possible, we highlighted potential molecular targets, the pros and cons of their targeting, and their potential therapeutic applications. We conclude that due to the immense complexity and the largely unexplored mechanisms of inflammation in atherosclerosis, effective molecular targets for developing therapeutic strategies are limited. Therefore, molecular inflammation in atherosclerosis is an emerging field with novel discoveries every year. Hopefully, we might 1 day be able to overcome these mechanisms and reduce inflammation in atherosclerosis, which could significantly alleviate the disease. Moreover, as described above, certain mechanisms are activated in macrophages, which could be promising for the prevention of atherosclerotic inflammation. However, further studies are required to substantiate the validity of these mechanisms and their therapeutic applications. Overall, we believe that expanding our knowledge of the basic molecular mechanisms and signaling cascades of inflammation in atherosclerotis should be prioritized to develop effective therapeutic interventions.

## Glossary

Chemotaxis: The enforced migration of immune cells such as monocytes/macrophages toward chemical stimuli, known as chemoattractants.
Classical monocytes: Highly phagocytic monocytes comprising over 90% of the plasma monocytes.
Efferocytosis: The phagocytosis of cellular debris by macrophages (specialized phagocytes) or other non-specialized cells.
Fibrous caps: Thick fibrous connective tissue in atherosclerotic plaques formed by smooth muscle cells beneath the endothelium, consisting of collagen, polysaccharides, and elastin.
Hypercholesterolemia: Refers to the excessively high levels of cholesterol in the blood, which is a risk factor for atherosclerosis.
Micropinocytosis: A process mediated by scaffold proteins like caveolin and clathrin, which form small membrane-derived vesicles to engulf macromolecules.
Myeloid cells: A type of blood cells produced in the BM then differentiated into diverse blood cells including macrophages, neutrophils, platelets, erythrocytes, eosinophils, and basophils.
Necrosis: A type of premature cell death mediated by autolysis.
Scavenger receptors: A superfamily of transmembrane receptors on innate immune cells with various functions such as lipid transportation and pathogen clearance.
